# Tactile, Auditory, and Visual Stimulation as Sensory Enrichment for Dairy Cattle

**DOI:** 10.3390/ani14091265

**Published:** 2024-04-23

**Authors:** Daniel Mota-Rojas, Alexandra L. Whittaker, Adriana Domínguez-Oliva, Ana C. Strappini, Adolfo Álvarez-Macías, Patricia Mora-Medina, Marcelo Ghezzi, Pamela Lendez, Karina Lezama-García, Temple Grandin

**Affiliations:** 1 Neurophysiology, Behavior and Animal Welfare Assessment, DPAA, Universidad Autónoma Metropolitana (UAM), Mexico City 04960, Mexicokikislezama@hotmail.com (K.L.-G.); 2 School of Animal and Veterinary Sciences, University of Adelaide, Roseworthy Campus, Adelaide, SA 5116, Australia; 3 Animal Health and Welfare Department, Wageningen Livestock Research, Wageningen University and Research, 6708 WD Wageningen, The Netherlands; 4 Facultad de Estudios Superiores Cuautitlán, Universidad Nacional Autónoma de México (UNAM), Cuautitlán 54714, Mexico; 5 Anatomy Area, Faculty of Veterinary Sciences (FCV), Universidad Nacional del Centro de la Provincia de Buenos Aires (UNCPBA), University Campus, Tandil 7000, Argentina; 6 Centro de Investigación Veterinaria de Tandil CIVETAN, UNCPBA-CICPBA-CONICET (UNCPBA), University Campus, Tandil 7000, Argentina; 7 Department of Animal Science, Colorado State University, Fort Collins, CO 80526, USA

**Keywords:** mechanical brushes, human–animal interaction, music, cattle behavior

## Abstract

**Simple Summary:**

Tactile, auditory, and visual stimuli have been proposed as enrichment types for dairy cattle to improve productive performance and overall welfare, including consideration of the mental domain. This review discusses the effects of mechanical brushes, tactile udder stimulation, music, and visual stimuli on dairy cattle welfare. The evidence suggests that mechanical brushes elicit positive emotional responses in cows. Music appears to have significant potential to improve welfare and productive performance. However, the type of music and tempo needs careful consideration. Other stimuli such as mirrors and pictures of conspecifics require further assessment to establish their impact.

**Abstract:**

Several types of enrichment can be used to improve animal welfare. This review summarizes the literature on the use of mechanical brushes, tactile udder stimulation, music, and visual stimuli as enrichment methods for dairy cows. Mechanical brushes and tactile stimulation of the udder have been shown to have a positive effect on milk yield and overall behavioral repertoire, enhancing natural behavior. Classical music reduces stress levels and has similarly been associated with increased milk yield. A slow or moderate tempo (70 to 100 bpm) at frequencies below 70 dB is recommended to have this positive effect. Evidence on the impacts of other types of enrichment, such as visual stimulation through mirrors, pictures, and color lights, or the use of olfactory stimuli, is equivocal and requires further study.

## 1. Introduction

Current research on dairy cow welfare does not only consider stress-related events but emphasizes the importance of achieving positive welfare states [1]. This is relevant because daily routine procedures performed inside dairy systems can be perceived by the animals as aversive and elicit negative responses [2,3]. This can be prevented by providing elements that enrich their environment, promote positive affective states, and improve the physical and mental health of animals [1,4,5].

Environmental enrichment (EE) consists of enhancing animal’s environment by modifying or adding items that encourage natural behaviors that can lead animals to experience positive emotional states [2,6]. Several studies have shown that EE improves the ability of domestic animals to cope with stress and can also be associated with preferences over certain objects [7,8]. The most frequently applied type of enrichment in dairy cattle is food-related [9]. However, sensory stimulation as an EE is also a promising method designed to stimulate one or more of an animal’s senses [8,10,11].

Tactile stimulation using mechanical brushes (MBs) (rotatory or stationary) enhances natural behaviors such as grooming and oral manipulation and has yielded benefits on the physiological, behavioral, and productive performance of dairy cattle [12,13]. On the other hand, auditory stimulation using instrumental music has been shown to reduce stress levels and might be associated with positive emotions in cattle [14], but these positive findings results are not always replicated.

An important review of environmental enrichment for dairy cows was published in 2016 by Mandel et al. [2]. However, there have been a range of studies conducted in the intervening years. This review will cover studies published since 2016 as well as landmark older studies. In addition, it presents the neurobiological background to the use of various enrichment forms. The focus will be on forms of tactile, auditory, and visual stimulation.

## 2. Search Methodology

A search was performed of the Web of Science (including CAB Abstracts), Scopus, and PubMed databases. The keywords used were a combination of “dairy cattle”, “dairy cow”, “environmental enrichment”, “auditory enrichment”, “tactile enrichment”, “dairy cattle music”, “mechanical brush”, and “visual enrichment”. The selected articles were those that included the investigation of the physiological, endocrine, or behavioral outcomes in a group of dairy cattle that provided forms of auditory, tactile, or visual enrichment, compared with those in non-enriched groups. The effects on the productive performance of dairy cattle were also considered. Excluded articles were those that used enrichment methods in beef cattle or other ruminant species.

## 3. Tactile Stimulation in Milking Systems

**Figure 1:**
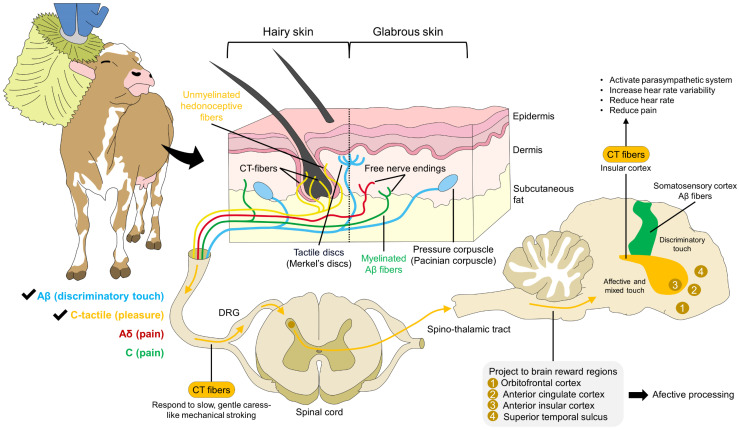
Neurobiology of affective touch. Gentle caressing or stroking activates low-threshold receptors. Although myelinated Aβ fibers are also related to discriminatory touch, in hairy skin, unmyelinated hedonoceptive fibers or C-tactile (CT) afferents process mechanical inputs associated with pleasure. CT fibers transduce and transmit this signal into the insular cortex through the spinothalamic tract. In the insular cortex, CT fibers activate the parasympathetic nervous systems, resulting in an increased heart rate variability and a decrease in heart rate and pain perception. Additionally, CT fibers project to the orbitofrontal, anterior cingulate, anterior insular cortices, and superior temporal sulcus, structures where the affective processing of touch is performed.

Tactile stimulation has been proposed to improve cattle welfare and elicit positive emotional states, derived from the knowledge that grooming is a biological need for cattle [15]. When animals cannot groom themselves, negative mental states (e.g., boredom) and abnormal behaviors (stereotypies) can arise [16,17,18,19,20]. Positive affective states linked to the use of tactile stimulation are related to the low-threshold receptors located in mammal skin and the so-called affective touch circuit that shapes and modulates the valence and response to tactile inputs (Figure 1) [21,22,23,24,25].

The implementation of MB—whether rotatory or stationary—inside dairy farms is an alternative that can have a positive effect on animal behavior and overall well-being, as discussed below.

### 3.1. Mechanical Brushes

Mechanical brushes are considered an essential part of EE in dairy farms because, in a natural environment, cattle use trees and other abrasive surfaces to scratch and groom themselves [2]. DeVries et al. [13] evaluated the implementation of MBs in 72 dairy cows. The brushes modulated the tine budget of cattle by increasing the total time spent scratching by 508% and the scratching frequency by 226%. Benefits were also found in cows provided with MBs in relation to maternal care. Enriched cows licked their calves for prolonged periods (8.7 min) in contrast to non-enriched cattle [13]. Similarly, Strappini et al. [26] determined in 25 weaned Holstein Friesian calves a preference (up to 60 times greater) for rotational MBs and horizontal rope when compared with stationary brushes. These results do not only support the idea that MBs motivate grooming but that cattle highly prefer spaces provided by such tactile stimulation.

The influence that MBs have on motivated behaviors was reported by McConnachie et al. [27] in pregnant and lactating Holstein cows. When comparing a room with fresh feed or one with an automated MB (where the animals could enter after pushing a weighted door gate), the authors found that cows were highly motivated to access the brush. Moreover, brushing was shown to encourage activity. Velasquez-Munoz et al. [15] reported that in 165 Holstein heifers, the addition of MBs decreases the not-active time by 1.1 ± 0.82 min. Additionally, eating time increased by 0.58 min/h, suggesting that grooming is a resource with a similar value as food.

MBs also have an effect on the emotional response of cattle. Oliveira and Keeling [28] studied the reaction of Swedish Red and Holstein dairy cows to three different events: feeding from individual roughage bins, using an MB, and queuing to enter a mechanical milking parlor. The authors used changes in facial expression (ear position) and body posture (neck and tail posture) to determine the valence and arousal provided by each stimulus. The findings showed that enriched cows having access to the MB maintained their ears backward up—or hanging—in an asymmetric position, with a horizontal neck, and a vigorously wagging tail. In contrast, queuing (regarded as a negative stimulus in non-enriched cows) resulted in ears pointing forward and upright, the neck below the horizontal plane, and the tail hanging without movement. The literature suggests that ears backwards or maintained horizontal and backwards (hanging ear posture), together with tail wagging, and a head above the horizontal plane, are indicative of a relaxed or excited state that can be enhanced by brush grooming [28,29,30].

Although MBs provide several benefits for dairy cattle, overall impact on animal welfare is influenced by other factors such as their social environment. For example, it has been observed that dominant cows use MBs more than submissive animals [31]. Foris et al. [32] also established that group size and facility design influence the use of MBs. In this study, Holstein cows increased usage of the MB (by 1.4 ± 0.5 min/day/cow) when it was placed near the feed and when cows were in small groups (by 0.5 h/day). Moreover, the suitable number of brushes that need to be provided according to the group size was studied by Reyes et al. [12] in Holstein dairy heifers. By placing two or four stationary brushes in groups of eight animals, grooming, oral manipulation, and displacement behaviors were recorded. The results showed that animals that were provided with more brushes used them for longer times (four brushes: 0.1 to 58.4 min, two brushes: 0.1 to 31.1 min). Nonetheless, total brush use was not affected, suggesting that animals tend to use the brushes because it helps them groom and satisfy this biological need.

Several authors agree that the use of MBs can reduce handling stress, observing a decrease in heart rate (HR) [13,18,19]. Likewise, the increase in the use of MBs after cows are separated from their calves reduces isolation and adverse reactions [19]. However, other studies have reported a non-significant effect on stress [17].

Therefore, since grooming is an essential behavior for dairy cattle, tactile stimulation through MBs can help motivate animals to perform species-specific behaviors. Additionally, cattle can also experience positive emotional states such as pleasure, elements that improve the level of welfare and their mental state [33,34].

### 3.2. Tactile Teat Stimulation

Tactile stimulation provided to dairy cattle is not limited to using brushes. The benefits of this type of sensory stimulation are also used to improve cows’ health and productive performance. Tactile teat stimulation is known to improve milk ejection, as mentioned by Bruckmaier and Wellnitz [35] in Holstein cattle. They observed that with tactile stimulation, milk ejection occurs at 40 s^−2^ min. This suggests that udder pre-stimulation enhances milk ejection. Nonetheless, apart from providing tactile enrichment, comfort in the milking parlor is necessary for optimal milking.

The physiological explanation for this effect is that during tactile stimulation of the udder, the activation of mechanoreceptors present in the mammary gland improves the venous return and reduces the release of pro-inflammatory substances (e.g., substance P) [36]. These receptors are also linked to the autonomic nervous system, reducing stress levels and promoting oxytocin release in the pituitary gland [37,38].

As with MBs, tactile teat stimulation also has contradictory results. Emamjome et al. [39] evaluated the effect of teat stimulation on the immune response of 14 Friesian dairy cows that received udder massage twice a day for four weeks. They observed that udder massage did not have a significant effect on the number of somatic cells or milk yield. The authors concluded that prolonged massage might be perceived as stressful, resulting in a negative reaction by the animals. Thus, it is necessary to carry out further research on the use of massage as a form of enrichment, and also on what type of stimulus can have a positive effect on dairy cattle.

## 4. Auditory Stimulation in Dairy Cows

**Figure 2:**
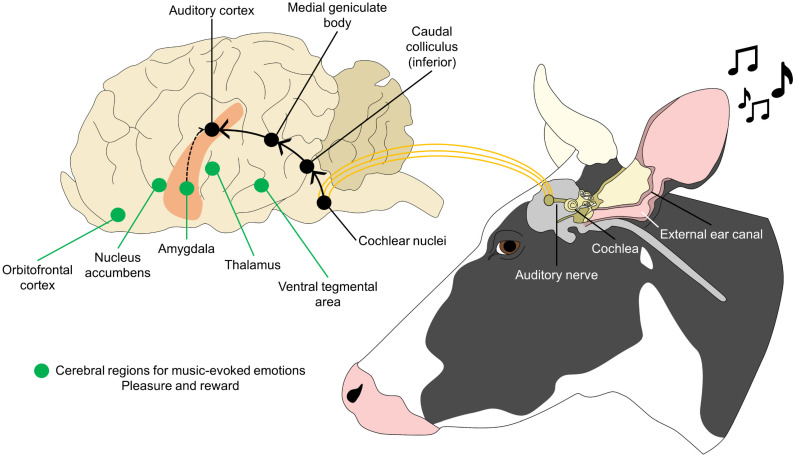
Neural pathways of music-evoked emotions. The emotional processing and benefits of music therapy depend on the connection between peripheral structures (e.g., cochlea and the auditory nerve) and central processing regions. After detecting the auditory stimulation, the auditory nerve projects to the cochlear nuclei to consequently transmit the stimulus to the auditory cortex. Conversely, the auditory cortex connects with cerebral regions that process music-evoked emotions such as pleasure and reward. Due to this neural pathway, music is suggested to elicit positive mental states in animals and reduce stress. Solid arrows represent the neural pathway from the cochlear nuclei to the auditory cortex; dashed arrows mark the connection between the amygdala and the auditory cortex.

Auditory stimulation through music has been used as an enrichment method to reduce the level of stress in livestock, particularly because environmental noises above 70 dB are considered stressful for dairy cattle [40], and maximum values of 96.10 dB at 500 Hz have been reported in milking parlors [41]. In humans, the beneficial effect that music has at a physiological and psychological level decreases anxiety, pain, stress, blood pressure, and heart rate [42,43,44]. It has been reported that listening to music has positive effects on social stress by reducing cortisol levels and even increasing the intensity of positive emotions due to the activation of cerebral structures related to emotions such as pleasure (Figure 2) [45,46,47,48,49,50]. In animals (e.g., pigs, dogs, chickens, buffaloes, and laboratory animals) [51,52], music has shown beneficial effects by reducing aggressive behavior, stress, and anxiety, considering it a sensory enrichment [53,54,55,56].

In dairy cattle, Uetake et al. [57] evaluated the effect of country music on voluntary approaches of lactating Holstein cows. The behavioral findings showed that the number of cows in the holding area of the milking parlor increased with music (from 22.3 ± 15.1% to 45.0 ± 18.0%), suggesting a higher readiness of cows to access the milking parlor. This coincides with what was reported by Crouch et al. [58], who compared the effect that classical music, country music, and audiobooks have on the behavior of 70 Holstein Friesian dairy cattle. It was found that all types of auditory stimuli significantly reduced the frequency of irregular behaviors such as tongue rolling or vocalization by 50%. Additionally, classical music and audiobooks stimulated positive social interaction between peers. These results show the relaxing effect that music can have on animals, reducing their stress level and possibly increasing productivity, but that periods without auditory stimulation might be beneficial as well.

Music can have a positive effect on the welfare of dairy cows and can be a useful tool to support daily milking routines. Erasmus et al. [59] observed in Holstein cows exposed to constant classical music that fecal glucocorticoid levels decreased (*p* = 0.012) while milk yields increased (*p* < 0.0001). In Vrindavani crossbred cows exposed to Indian instrumental music (with flute and sitar), no differences were found between enriched and control animals in terms of milk yield (12.57–12.85 kg), rectal temperature (99.82–100.08 °F), respiratory rate (22.2–23.47 counts/min), and thyroid hormones. However, lower values were found in enriched cows for cortisol (49.11–61.85 nm/L vs. 84.45 nm/L), milking time (10.67–10.70 min vs. 10.98 min), milk flow rate (1.19–1.21 kg/min vs. 1.16 kg/min), and kicking behavior (61.31–63.44% vs. 71.72%). Consequently, the authors concluded that music can reduce residual milk by improving the welfare of dairy cows during milking [60].

In Holstein Friesian, Jersey, and Ayrshire cows, Lemcke et al. [61] selected 57 pieces of blues, rock, and classical music with a tempo of less than 100 bpm to combine them into a single playlist to be played continuously for 48 h. The frequency ranged from 200 to 16 kHz with an amplitude of 65–70 dB. Although no differences were observed for dairy milk yield and milking interval between animals with and without music, milking frequency (3.0 vs. 2.8) and gate passing frequency (15.8 vs. 13.8) were higher in enriched cows. In crossbred cows, exposure to classical music with a slow (75–107 bpm) and moderate tempo (90–100 bpm) resulted in increased daily milk production (by 50–65%) and reduced residual milk (by 23.80–24.60%) when compared to control animals [62]. Moreover, in comparison with the control group, cows receiving auditory enrichment had higher serotonin levels (385.67 vs. 213.625 ng/mL), a neurotransmitter associated with positive mental states.

Jiajia et al. [63] found that mellow light music (at 70 bpm) improved lactation (nutrient intake and milk yield) performance in Holstein cows. In contrast, Shamshul and Yusof (2023) [64] concluded that music did not improve the milk yield of Jersey dairy cows when cows were exposed to a single playlist (Clayderman’s classical piano and Mozart’s classical music) maintained at a sound pressure level below 85 dB and frequencies ranging between 23 and 35 kHz. Likewise, Kıyıcı et al. [65] did not find significant differences in milk yield in Holstein Friesian cattle exposed to classical music (10.87 ± 0.18 L) and control cows (10.83 ± 0.20 L).

As music can have beneficial effects, the presence of loud noise can significantly alter livestock [66]. Ciborowska et al. [14] explained that if dairy cattle are exposed to prolonged periods of high-frequency noise (with a threshold point of sound of 37 kHz), this can negatively affect the quality and quantity of milk yield, which can result in economic losses for the producer. These authors suggest alternating auditory stimuli with periods of silence. Contrary to this, conditioning to sounds may engender positive responses to routine events, for example, conditioning cows to country music on milking parlor entry encourages forward movement and improves animal handling [57]. This means that, before considering music as sensory enrichment, the type and duration of the stimulus need to be considered.

Auditory stimuli such as music can be used in cattle as a way to reduce the level of social stress that these animals might experience during milking. Nonetheless, today, there is no validated type of music/sound regarded as positive as the reaction may be influenced by the preference for some types of auditory stimulus.

## 5. Visual Stimulation in Dairy Cows

Ruminants need to maintain visual contact with conspecifics and social grouping to reduce distress by isolation and attenuate stress-mediated responses (e.g., increase in plasma cortisol and glucose) [67,68]. Their binocular vision and ability to identify pen peers are also important as they are gregarious animals that require social interaction [17]. This derives from the protection that animals might feel when surrounded by conspecifics and the strong preferential bonds that cattle can establish early in life [67].

**Figure 3:**
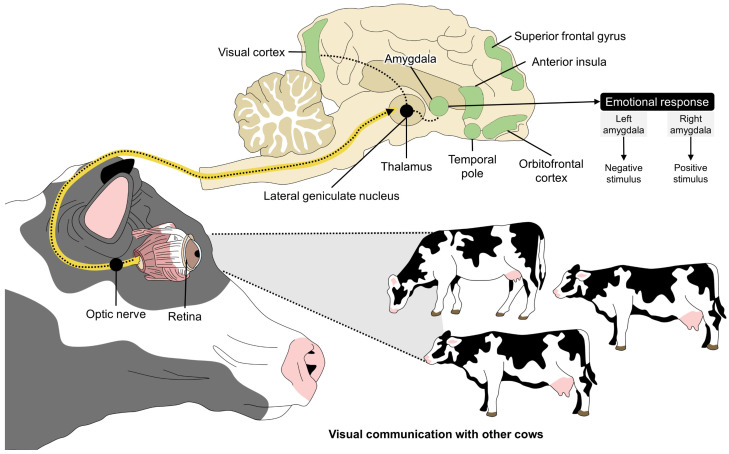
Neurobiological processing of visual stimuli. When animals are exposed to a visual stimulus (positive/negative), the main visual pathway connecting the retina and optic nerve to the lateral geniculate nucleus and, finally, the visual cortex, is the first step to recognizing an object (e.g., the presence of a conspecific). The lateral geniculate nucleus of the thalamus also shares projections with the amygdala, the main center that integrates emotional responses. Other regions such as the temporal pole, orbitofrontal cortex, superior frontal gyrus, and the anterior insula have been identified as structures that activate when perceiving visual stimulations.

Cattle are social animals that perceive isolation as an aversive stimulus [69]. Therefore, to avoid the consequences of social isolation in this gregarious species, adding elements such as mirrors or conspecific pictures has been proposed to reduce fear and isolation stress due to the effect that visual stimulation has on the emotional state of animals (Figure 3) [17,70,71,72,73]. An example is the study by Piller et al.’s [70], in which Black Angus heifers were exposed to frontal or side-view mirrors when confined to a weigh scale. According to the HR and amount of movement, heifers in the front-side mirror group had lower average HR than those in the side-view mirror group (91.9 ± 1.9 bpm vs. 98.0 ± 2.0 bpm) and moved less (34.8 ± 4.1 and 68.9 ± 6.6 moving-measurement device). This can be interpreted as a reduced response to isolation. In another study, Mandel et al. [17] used mirrors as visual enrichment during husbandry procedures that require isolation (e.g., artificial insemination, claw trimming, and medical treatment). However, no improvements in HR (control: 67.8 bpm vs. mirror: 67.3 bpm), heart rate variability (HRV) (control: 10.7 vs. mirror: 10.6), or behavioral responses associated with stress (e.g., frequency of vocalization, locomotion, exploring, etc.) were reported.

Although studies using mirrors as visual enrichment for dairy cattle are limited, another alternative to reduce fear behaviors is the use of images from conspecifics to simulate social interaction. This was studied by Ninomiya and Sato [74] in four socially isolated Japanese Black cows. The authors compared the reaction of the animals to a blank picture, a picture of a familiar cow in actual size, an unfamiliar Holstein cow picture, and the actual presence of a companion cow. By assessing vocalization and salivary chromogranin A and cortisol concentrations, it was found that at 30 min of isolation, the chromogranin concentrations were lower in cows where a familiar cow picture was displayed (8.9 ± 1.6 pmol/mL) and in the presence of a companion cow (11.8 ± 9.3 pmol/mL), in contrast to the cows exposed to a blank sheet (28.1 ± 14.4 pmol/mL). Similarly, the salivary cortisol concentrations were lower in cows exposed to a picture of a familiar cow and to the presence of a conspecific (0.33 ± 0.19 pmol/mL and 0.22 ± 0.09 pmol/mL). Likewise, the number of vocalizations was lower, with 3.7 and 0 vocalizations, respectively. These results show that displaying a picture of a familiar conspecific might reduce stress in cattle for a short period. Nonetheless, the actual presence of a conspecific seems to have more benefits.

In another study from the same authors, the preference of Japanese Black cows for a brush or a picture of a peer’s face of the actual size of a cow’s face was evaluated. It was found that animals stayed longer (average of 273.4 min) in the pen with the picture and sniffled the face picture an average of 8.1 times, in contrast to control animals (174.8 min) and those provided with a brush (213.1 min) [75]. These results are interesting and show that ruminants prefer to be with familiar conspecifics and avoid social isolation as a part of their gregarious nature [76].

Nonetheless, it must be emphasized that providing visual stimulation in the form of pictures or mirrors lacks tactile, auditory, and olfactory cues that would be found in a real conspecific [77]. Therefore, providing them might have limitations, as seen in the available studies where visual stimulation does not always represent an advantage for cattle.

Although research in adult dairy cattle interacting with other individuals is limited, the benefits of social interaction with conspecifics can be seen in milking parlors. On the one hand, in highly social species, the so-called “social buffering”, a phenomenon where interaction with conspecifics results in reduced stress- and fear-related responses, has a positive influence on the physiological and psychological well-being of animals [67,77]. The effect of social buffering has been studied during painful situations such as disbudding. This was reported in Holstein Friesian dairy calves when comparing pair vs. individual housing for hot iron disbudding with local anesthetic. The authors reported that pair-housed calves increased feeding behavior during and before disbudding (Ismeans: 8.23 and 8.41, respectively). Nonetheless, pain-related behaviors such as head shaking, head rubbing, foot stamping, and self-grooming did not have differences between both groups [78].

Furthermore, securing calf presence to increase milk yield and reduce aggressive behaviors in cattle has also been proposed for dairy cattle. For example, Tancin et al. [79] recorded oxytocin release during milking with and without calf presence in Brown Swiss cows. The authors found that milking during the presence of calves resulted in higher oxytocin release (between 10.6 ± 2.8 pmol/l and 24.4 ± 5.3 pmol/l) and milk yield (11.9 kg vs. 10.9 kg) than when calves were removed. However, during suckling by their own calf, the oxytocin concentrations were higher (between 42.1 and 43.4 pmol/l). In cases where calf presence was not possible, Zipp et al. [80] used played-back calf calls, hair of the calf, and teat massage as the auditory, olfactory, and tactile stimulation of cows. However, although milk yield was higher in cows where calf contact was allowed within milking times (+9.9 kg/milking), no improvements in agitation behavior, HR, or HRV were found.

Similarly, in another study by the same authors, hair from their own calf or an alien calf was used to record the behavioral response of German Red Pied and German Holstein black-and-white cows. Although 60% of cows reacted to both stimuli, a higher response (sniffling or licking) was detected with the odor of their own calves (own: 37.5%; alien: 25%) [81]. These results suggest that adding calf-related sensory inputs can help cattle recognize familiar conspecifics, but it has a limited effect on dairy cattle [81]. Nonetheless, acknowledging that cattle require interaction with conspecifics, displaying pictures or using additional sensory stimulation might be types of EE that require further study.

## 6. Opportunities to Improve Sensory Stimulation during Milking

The evidence discussed indicates that multisensory stimuli such as tactile, auditory, and visual stimulation can promote positive emotional states that reduce the level of physical and social stress. The addition of other elements such as ropes is still ongoing. An example is Miranda et al. [82], who evaluated the effect of brushes and ropes on behavioral and physiological variables in 18 Gir x Holstein calves during toothbrushing (an aversive event). In the enriched animals, the handling duration was 0.54 ± 0.08 min shorter than non-enriched cows. Although no effects on cortisol and lactate were observed, the authors concluded that low-cost EE could facilitate management. On the other hand, providing a supplemental lighting system with three different colors (white, yellow-green, or blue) to Holstein cows while lying down is another field where EE might have benefits [83]. In Wilson et al.’s [83] study, the three different lighting colors did not affect lying time (white and no supplementation: 6.5 ± 0.71 h/d; white and yellow-green: 6.0 ± 0.61 h/d; and white and blue: 6.2 ± 0.58 h/d) with an average of 4.5–4.8 bouts/d. These results suggest that cows do not avoid LED light but that evaluating other parameters should be addressed.

Rotating techniques of enrichment (e.g., one different item each week) have also been proposed as a way to improve the housing environment in Holstein dairy farms. This was reported by Zhang et al. [84], who compared a control group of calves without enrichment, a rotatory group, and a fixed enrichment group from four to seven weeks of age. The items were stationary brushes, ropes, springs, nets filled with strawberry-scented hay, and dry treats. It was found that calves with fixed enrichment interacted more often with the items than the other two groups (fixed: approximately 49%; rotatory: ~20%), especially with the strawberry-scented hay (2.0–2.5% of the scans). This study also shows the importance of providing olfactory stimulation to dairy animals.

Limited studies have been performed regarding olfactory stimulation in dairy cows (and in livestock in general). Abd Rahim et al. [85] tested an artificial hay aroma to evaluate the effect on milk yield in Holstein Friesian cows to promote voluntary hay intake. The addition of the artificial aroma increased compound feed intake (18.0–19.2 kg) and total dry matter intake (22.7–24.0 kg) but did not improve milk yield or milk composition. Other studies have concluded that even predator odor (brown bear) does not reduce milk yield when compared to other odors (bear: 24.8 ± 4.4 L; red deer: 24.2 ± 4.6 L; and blank: 24.4 ± 5.1 L) [86]. In horses, research using essential oils (e.g., vetiver, spikenard, roman chamomile, lavender) to evaluate their calming effects has been studied [87]. However, future studies need to explore this in livestock to determine their effect on dairy cattle welfare and milk yield. In dairy calves (mainly Swedish Holstein and Swedish Red breeds), sensory enrichment through an attractive odor (freshly cut grass) and taste (glucose-coated teat) was used to evaluate if suckling behavior is enhanced. The grass odor had no significant effect on suckling behavior. In contrast, calves had a preference for glucose-coated teats, spending more time on the enriched teat (50.67 s) than the control one (30.56 s) [88]. Nonetheless, to date, research towards which odors evoke positive reactions in dairy cattle is still ongoing.

Finally, considering the “emotional contagion” process that occurs when the affective state of one animal is influenced by the perception of the affective state of another individual, relevant for gregarious species such as livestock, is also necessary to evaluate the effect of sensory enrichment not only in individuals but the effect of the stimuli on the herd and its social impact, as mentioned by Baciadonna et al. [89].

## 7. Conclusions

Current dairy farming is focused on improving livestock welfare to reduce the impact that potential stressors might have on the physical and mental health of animals. Sensory enrichment through tactile, auditory, and visual inputs facilitates species-specific behaviors, a trait essential to elicit positive mental states in livestock. Providing rotatory and stationary mechanical brushes to dairy cattle has been shown to enrich their behavioral repertoire and promote relaxation. On the other hand, using classical music or soundtracks with a slow or moderate tempo (70 to 100 bpm) at frequencies below 70 dB seems to improve the behavior and lactation performance of dairy cows and contribute to positive mental states.

Other types of sensory enrichment such as visual stimulation suggest that displaying pictures of conspecifics pictures when cattle need to be isolated for certain periods reduces stress-related responses. Nonetheless, studies have concluded that the actual presence of conspecifics provides more benefits, such as the ones observed in lactating cows when in the presence of their calf during milking.

Alternative sensory enrichment using different types of light colors, adding items to the pens, or olfactory stimulus is still ongoing. However, enriching the environment of dairy cattle and stimulating the animal’s senses is a method used to encourage natural behaviors and positive emotional states.
